# Processing of a Subliminal Rebus during Sleep: Idiosyncratic Primary versus Secondary Process Associations upon Awakening from REM- versus Non-REM-Sleep

**DOI:** 10.3389/fpsyg.2017.01955

**Published:** 2017-11-20

**Authors:** Jana Steinig, Ariane Bazan, Svenja Happe, Sarah Antonetti, Howard Shevrin

**Affiliations:** ^1^Department of Psychosomatic Medicine, University of Leipzig, Leipzig, Germany; ^2^Institute of Psychology and Cognition Research, University of Bremen, Bremen, Germany; ^3^Department of Clinical Neurophysiology, Klinikum Bremen-Ost, University of Göttingen, Göttingen, Germany; ^4^Faculté des Sciences Psychologiques et de l’Education, Service de Psychologie Clinique et Différentielle, Université Libre de Bruxelles, Brussels, Belgium; ^5^Department of Neurology, Klinik Maria Frieden, Telgte, Germany; ^6^Department of Psychiatry, University of Michigan Medical Center, Ann Arbor, MI, United States

**Keywords:** primary process, Freud, rebus, dream, free association, subliminal, REM

## Abstract

Primary and secondary processes are the foundational axes of the Freudian mental apparatus: one horizontally as a tendency to associate, the primary process, and one vertically as the ability for perspective taking, the secondary process. Primary process mentation is not only supposed to be dominant in the unconscious but also, for example, in dreams. The present study tests the hypothesis that the mental activity during REM-sleep has more characteristics of the primary process, while during non-REM-sleep more secondary process operations take place. Because the solving of a rebus requires the ability to non-contexually condensate the literal reading of single stimuli into a new one, rebus solving is a primary process operation by excellence. In a replication of the dream-rebus study of Shevrin and Fisher (1967), a rebus, which consisted of an image of a comb (German: “Kamm”) and an image of a raft (German: “Floß”), resulting in the German rebus word “kampflos” (Engl.: without a struggle), was flashed subliminally (at 1 ms) to 20 participants before going to sleep. Upon consecutive awakenings participants were asked for a dream report, free associations and an image description. Based on objective association norms, there were significantly more conceptual associations referring to *Kamm* and *Floß* indexing secondary process mentation when subjects were awakened from non-REM sleep as compared to REM-awakenings. There were not significantly more rebus associations referring to *kampflos* indexing primary process mentation when awakened from REM-sleep as compared to non-REM awakenings. However, when the associations were scored on the basis of each subject’s individual norms, there was a rebus effect with more idiosyncratic rebus associations in awakenings after REM than after non-REM-sleep. Our results support the general idea that REM-sleep is characterized by primary process thinking, while non-REM-sleep mentation follows the rules of the secondary process.

## Introduction

In the first pages of his “Project for a scientific psychology,” Freud (1895/1950) proposes two founding axes upon which the whole architecture of his mental apparatus is resting, namely the primary and secondary processes – respectively, a horizontal axis as a tendency to associate and a vertical one as the ability for inhibition and perspective taking. Indeed, primary process thinking is characterized by mechanisms of condensation, displacement, substitution, compromise-formation and superficial associations as well as “faulty reasoning, absurdity, indirect representation, representation by the opposite” (Freud, 1905/1960, p. 88–89). It prevails in the unconscious, where its manifestations are thought to be hallucinatory, unrealistic, not time-bound, and irrational. Therefore, it prevails in all different kinds of phenomena which reveal close relationships to unconscious processes, such as neurotic and psychotic symptoms, slips of the tongue and other parapraxes, jokes, transference manifestations, fantasies and free associations, as well as altered states of consciousness, such as sleep, dreams or hypnosis. The secondary process functions to inhibit and control primary process tendencies (Freud, 1900/1953; Bazan, 2012). It therefore allows to disengage from a direct reaction upon the stimulus enabling the organism to have a perspective upon the situation. This process is said to be “attuned to the efficient attainment of goals in reality with the delayed gratification of impulses that is necessary” (Holt, 2009, p. 3). This “more sophisticated” (Holt, 2009, p. 3) secondary process gains in importance and fully develops in the course of life. It is an ordered and goal-directed thought process – mostly logical, rational, non-hallucinatory, self-correcting and realistic, which can be found in our awake and conscious thinking.

Other authors after Freud have further elaborated the concept of primary process. Rapaport proposes that the primary process is characterized by the toleration of contradictions, “omnipotence of thought [and] pars pro toto” (Rapaport, 1951, p. 694), Holt speaks about an association on the basis of “non-essential” features (Holt, 1967, p. 354). More recently, Roussillon has qualified the primary process as the locus of the “everything, all at once, all alone, all together, all in one” (Roussillon, 2007, p. 33). As concerns empirical evidence, in the team of Shevrin (1973), Brakel et al. (2000, 2002) showed that primary process similarity judgment is based upon a commonality of attributes of the stimulus (elements with the same forms) and Shevrin and colleagues have shown that this is especially the case for *linguistic attributes*, such as common phoneme sequences (e.g., rebuses, see also Shevrin and Fisher, 1967; Klein Villa et al., 2006).

The primary and secondary process, far from being an old-fashioned pair of concepts, reveals to be one of the liveliest and most generative Freudian concepts. Not only have they led to the development of a variety of tools for their measurement – some of which close to the psychoanalytic practice (e.g., Martindale and Dailey, 1975, 1990; Holt, 2009), others close to cognitive categorisation theories (Brakel et al., 2000) – they also have led to fruitful neuroscientific reinterpretations. Bazan (2007a,b) proposes a parallel between the primary process and the content-treating so-called ventral ‘What’-pathway and between the secondary process and the spatial, inhibitory so-called dorsal ‘Where’-pathway. This model is compatible with the model of Carhart-Harris and Friston (2010), who propose that in non-ordinary states of consciousness, such as acute psychosis, dreaming and hallucinations, there is a failure of the top-down inhibition, which normally controls the so-called *default-mode network* (DMN),, whereby a limbic overactivation of this neocortical DMN is not countered – this DMN exacerbation then resulting in primary process mentation.

However, it is important to note that there is no pure primary or secondary process but rather a continous interplay between these two modes of mental functioning (Rapaport, 1951; Gill, 1967). Hence, primary and secondary process thinking do in fact take place simultaneously but – depending on the respective state of consciousness, for example –one or the other prevails. As there are many different states of consciousness ranging from the most vigilant waking state to the deepest sleep stage, thinking is supposed to be increasingly taken over by primary process thinking including transformative and more dreamlike ways of processing information, the more one descends levels of consciousness (Rapaport, 1951; Holt, 2009).

Both dreams, which are supposed to be elicited by unconscious and repressed wishes, and dream work, which functions to convert the latent dream thoughts into the often bizarre language of the manifest dream content, are especially characterized by primary process mechanisms (Freud, 1900/1953). Day residues offer the material for more existential wishes to elaborate upon, but as the motor execution pathway is blocked during sleep, there is a regression toward representational activity (Solms, 1995, 1997). This representational activity is based upon the original *linguistic* storyline of these wishful thoughts and not directly upon images: the dream searches to find images to express abstract concepts, names, or even grammatical operations (Freud, 1900/1953). For an example of an abstract concept, Freud (1900/1953, p. 212), in an analysis which he carried out in French, had to interpret a dream in which he appeared as an elephant. Upon asking the dreamer he answered: “Vous me trompez” (Engl.: “You betray me”). Thereby the dreamer used the representable image of an elephant to express the abstract concept of “betrayal,” since the French word *trompe* both has the meaning of “trunk” and “to betray.” For an example of a name, Freud cites Tausk (1914) who describes a short dream fragment where the dreamer sees a girl on the road to X bathed in a white light and wearing a white blouse; it appeared the dreamer began an affair with a Miss White on this road. Again, the dreamer used the representable image of a white dress to express the name of Miss White since the word “white” both denotes a color and a person. When the wish is representable and not defended against, it is thought to be directly expressed in the images of the dream (see e.g., Anna Freud’s strawberry-dream; Freud, 1900/1953). However, when the wish is either difficult to represent in images and/or the wish has to be defended against, then the polysemy^1^ of the words is used at its greatest advantage to produce the dream images, pushed by the regression toward representation.

For Freud, indeed, the dream is a rebus and “our predecessors in the art of dream-interpretation have made the mistake of judging the rebus as an artistic composition” (Freud, 1900/1953, p. 170)^2^ Shevrin et al. (1996, p. 107) claim that rebuses investigate “a formal aspect of dynamic unconscious thought organization^3^ marked primarily by superficial associations in the form of phonetic transitions and combinations.” Names are treated as objects and are condensed in new, sometimes bizarre word creations. This, by excellence, is a primary process dynamic as it does not take the context into account. Among the shorter dream fragments from Freud’s epical *Interpretation of dreams*, here is one which illustrates the rebus principle: a patient relates a dream in which his uncle gives him a kiss in an automobile and the patient immediately adds the interpretation: “It means auto-erotism” (Freud, 1900/1953, p. 127). We may add some short fragments from our own clinical practice (second author; clinical practice mostly in Dutch): (1) an English speaking woman dreamt she was sitting facing her therapist while the soles of their feet were touching; the meaning of this bizarre fragment became clear when she described the scene (in analysis) as “we were sitting sole to sole”; (2) a woman, upon coming back from South-Africa, dreamt she was paying in a bar with white pieces of paper upon which a square was drawn near the borders; she thus was paying with four-edged bills, i.e., with “rand”-s, *rand* being both the Dutch word for edge and for the South-African currency; (3) a woman dreamt she was writing a letter and could not complete the two final lines of the letter, the Dutch word for lines she used being the ambiguous word *regels*, which means both lines and periods; the patient suddenly understood she feared she was pregnant as she twice forgot to take contraception that month (see also Bazan, 2007b, pp. 20–21 for more examples). What Freud tries to point out is that the dream should not be taken metaphorically, which is a secondary process operation, but literally on the grounds of primary process logics^4^: free associations, devoid of their grammatical constraints and thus available for the exploration of their proper polysemy, are used to trace the way back from the manifest dream content to the latent dream thoughts, which are thought to testify of the underlying unconscious wishes. Hence, if dreams result from the disguising mechanisms of dream work through an unhibited, associative way of thinking – the primary process – dreams might be particularly suitable for the investigation of the nature of primary process thinking.

Against former assumptions, dreams do not only occur during rapid-eye-movement-sleep (REM-sleep), but also during non-REM-sleep (Solms, 2000), although the average REM dream report rate is much higher as compared to the average non-REM dream report rate (Nielsen, 1999). Still, there are important qualitative and quantitative differences: while REM-sleep dreams are mostly longer (Foulkes and Rechtschaffen, 1964; Antrobus, 1983; Stickgold et al., 1994), more bizarre (Fiss et al., 1966; Porte and Hobson, 1987; Zepelin, 1989), more vivid, visual, emotional, and less related to waking reality (Foulkes, 1962; Rechtschaffen and Kales, 1968; Cavallero et al., 1992), non-REM-sleep mentation is usually shorter, less imaginative, bizarre and emotional, more thought-like, and consists mostly of single thoughts or ideas often related to waking life (Foulkes, 1962; Rechtschaffen and Kales, 1968). We propose to understand these differences between REM- and non-REM dream descriptions in terms of differences between primary and secondary processing and hence, what we propose is that, even if dreams are reported both after REM- and non-REM-sleep awakenings, only REM-dreams follow the primary process logic as described by Freud. This is to say that, in terms of the present operationalisation, only in REM-dreams will we find traces of a rebus reading of pictorial stimulus material presented just before sleep.

The only study, which tried to answer this particular question, was run by Shevrin and Fisher (1967) who used a rebus, namely a stimulus which consisted of the picture of a pen and a knee, forming together the rebus word “penny.” Shevrin and his co-workers were able to demonstrate that only the subliminal presentation – and therefore unconscious processing – of the rebus stimulus leads to answers and associations related to the rebus word while this was not the case after supraliminal – and therefore fully conscious – presentation (Shevrin and Luborsky, 1961). The term subliminal derives from the Latin words *sub* (Engl.: under) and *limen* (Engl.: threshold). Following a definition by Merikle « subliminal perception occurs whenever stimuli presented below the threshold or limen for awareness are found to influence thoughts, feelings or actions » (Merikle, 2000, p. 497). The first to demonstrate that subliminal presentation of a stimulus leads to unconscious processing and reworking of this stimulus was Otto-Poetzl (1917). Especially in the 1950s the method of subliminal stimulation was used to investigate unconscious processes (e.g., Fisher, 1956; Luborsky and Shevrin, 1956; Shevrin and Luborsky, 1958; Klein, 1959; Fiss et al., 1963). Using the subliminal method, Shevrin and Fisher (1967) found answers referring to the rebus stimulus only after awakenings from REM-sleep, but not after non-REM-sleep awakenings. However, this early study suffered methodological limitations, like a rather small sample size (*N* = 10) and less rigorous subliminal conditions (presentation time of 6 ms and no detection experiment to assure subliminality) and there has been no further supportive evidence for its findings so far. Hence, the purpose of this study is to replicate this Shevrin and Fisher study and thereby test the hypothesis that the mental activity during REM-sleep has more characteristics of the primary process, while during non-REM-sleep more secondary process operations take place. As in the original Shevrin and Fisher study, a waking rebus will be presented just before sleep and traces of either rebus or conceptual associations on the stimulus material will be measured in the production of the participants. In contrast to the original study, we will work with shorter presentation times (1 ms instead 6 ms) and with experimental conditions controlled for stringent subliminality. Snodgrass and Shevrin (2006; see also Bazan, 2017) propose that only at these extreme conditions of subliminality one is able to tap directly into the dynamical unconscious as shown by different empirical studies (e.g., Klein Villa et al., 2006). If primary processes prevail during REM- and secondary processes during non-REM-sleep, we are to find more rebus associations upon REM-sleep awakenings and more conceptual associations to the stimulus material upon non-REM-sleep awakenings.

## Materials and Methods

### Subjects

Twenty native German speaking university students (5 male, 15 female) were recruited by postings on campus of the University of Bremen. Subjects ranged in age from 20 to 35 (mean age 23.6 ± 3.2 years) and met the following inclusion criteria: no self-reports of diagnosed sleep disorder, of a history of neurological or psychiatric illness, of current medication use or of drug or alcohol abuse. Self-reported sleep efficiency (total sleep time/time in bed) ranged from 78 to 99% (90.6 ± 6.6%) and average total sleep time from 6 to 10 h (8 ± 1.3 h). 90% rated their sleep quality as “good” (50%) or “very good” (40%). Indeed, all subjects had a good sleep and a healthy sleep architecture, as displayed in the baseline night in the sleep lab prior to the experimental nights (see below). Subjects received 70€ when they completed the study. The study protocol was designed according to the Helsinki Declaration (1964/2004) and approved by the ethics commission of the University of Bremen. All subjects provided written informed consent prior to the study.

### Protocol

Subjects spent three nights in the sleep lab. The first night was the baseline night. By this, the subject could get used to the environment of the lab and to sleeping with the electrodes. At the same time, a healthy sleep cycle could be assured and sleep disorders of any kind ruled out (i.e., obstructive sleep apnea syndrome, periodic limb movement disorder, somnambulism, REM-sleep behavioral disorder, etc.). With an interval of 5–7 days respectively, followed the experimental night and the control night. The order of the experimental and the control night was randomized and counterbalanced. Both, the experimenter and the subject, were blind to the respective condition.

### Sleep Stage Recordings

Polysomnographic recording included four scalp electrodes (C3, C4, O1, O2) with contralateral mastoid references, following the international 10–20 electrode placement system (Jasper, 1958), bipolar electrooculogram and bipolar submental electromyogram. Visual scoring of wakefulness and sleep stages was done following the standardized rules of Rechtschaffen and Kales (1968), using 30s epochs for scoring. Data was recorded using Excel-Tech (XLTEK) hard- and software. To detect possible sleep disorders, anterior tibialis leg electromyography, electrocardiogram, as well as measures of airflow, respiratory effort, and blood oxygen saturation was additionally used in the baseline night.

### Rebus Stimulus

**FIGURE 1:**
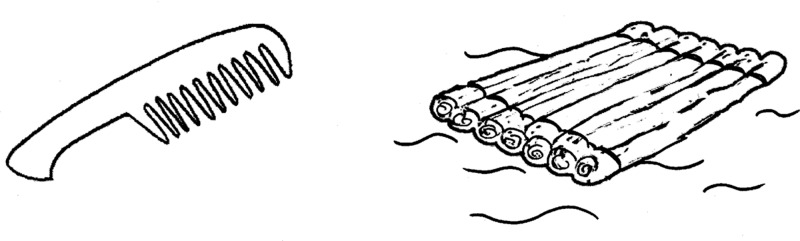
The “kampflos” rebus stimulus. The rebus stimulus consists of the two pictured objects “Kamm” (Engl.: comb) and “Floß” (Engl.: raft). The condensation of the phonemes of both objects leads to the rebus word “kampflos” (Engl.: without a struggle/fight).

For the investigation of primary and secondary process during sleep we used a rebus stimulus. Our rebus consists of the two pictured objects “Kamm” (Engl.: comb) and “Floß” (Engl.: raft; see **Figure 1**). The condensation of the phonemes of both objects leads to the rebus word “kampflos” (Engl.: without a struggle/fight). The conventional reading of this picture would result in the identification of the comb and the raft and therefore lead to secondary process associations on the so-called *conceptual level* referring to the pictured objects, for example hair, head, water, wood, etc. A primary process reading of the pictures, however, would lead to associations on the *rebus level* referring to the rebus *kampflos*, for example victory, defense, enemy, fight.

### Stimulus Presentation and Detection Experiment

In the experimental night, this rebus stimulus was presented subliminally before the subject’s retiring to bed. To control for the experimental effect of the stimulus, there was a control night in which a blank slide was presented instead of the rebus stimulus. Thus, each subject was his or her own control and a measure of base rate recovery could be provided (Dixon, 1971). Rebus and control stimulus were presented by an electronical projection-tachistoscope EPT 5a which consists of two Kodak carousel-projectors and an external control box. The projectors work with xenon high-pressure lamps which are characterized by very short rise and drop times of only 30 μs. In case of subliminal stimulation, where presentation times are supposed to be extremely short to avoid conscious awareness of the stimuli, this is a crucial factor. Projector 1 is switched off and flashes only during the defined presentation time to present the stimulus. Projector 2 is switched on and gets dark during this stimulus presentation. Because of this uninterrupted cross-fading the light is continuously on and the subject does not realize when the picture is being flashed.

Stimulus presentation was set at 1 ms and luminance at 5 fl (foot lamberts). The total number of presentations was 5 with time intervals of 1 s between each of the five flashes. The size of the projection screen was 100 × 66 cm, the picture of the *Kamm* and the *Floß* covered a square of 24 × 6 cm. The distance from subject to screen was 2.35 m. Subjects were asked to sit down in front of the screen and look at the fixation cross at the center of the screen. By saying “Ready!” just before flashing the stimulus five times, subjects were warned and knew when to focus on the fixation cross and not to blink their eyes. Either the rebus stimulus or a blank control slide was flashed, immediately followed by the reappearance of the fixation cross.

Working with this duration and luminance has proven to assure working at the so-called objective detection threshold (ODT) which represents the most stringent criterion for subliminality (Snodgrass, 2004; Snodgrass et al., 2004a,b; Snodgrass and Shevrin, 2006). To assure that the stimulus was indeed presented totally subliminally, a forced-choice detection experiment was run the morning after the last night in the sleep lab. Therefore, 32 cards with the rebus stimulus and 32 blank cards were flashed in randomized order under the experimental conditions described above. Subjects were asked to state after each presentation whether they had seen “something” or “nothing” and to keep their responses approximately equally divided between these two choices. To meet the conditions of the ODT, subjects must not be able to detect a difference between the rebus and the blank. If they see “something” – even if they cannot identify it as a comb or a raft – they are no longer at the ODT. Thus, if *d*′ as measure of conscious perception (sensitivity to discriminate between stimulus and blank) is at chance (*d*′ = 0), the subject cannot detect a difference between stimulus and no-stimulus and subliminality is guaranteed.

### Tasks

During the experimental night and the control night, subjects were awakened three times from REM-sleep (condition “REM”) and three times from non-REM-sleep stage 2 (condition “non-REM”). The first awakening was a non-REM awakening immediately following the first sleep cycle, and the last one was a REM awakening early in the morning. After each awakening, subjects were asked to perform three different tasks (see below). Two of these tasks (free associations and image description) were also obtained in the waking state right after stimulus presentation (condition “wake”).

#### Dream Report

Immediately upon awakening the experimenter asked, “What was happening before I awakened you?” and added “Please recall what was happening in as much detail and as elaborately as possible. Allow enough time to avoid forgetting something.”

#### Free Associations

After this, the subject was asked to close his eyes and to say all the individual words that came to mind for the following 4 min. The subject was explicitly instructed to say *all* words – no matter how related or unrelated, no matter how silly or non-sensical they might seem.

#### Image Description

Finally, the subject was asked to describe the first picture that came to mind and to make a drawing of it.

### Debriefing

The morning after the last night in the sleep lab, there was a debriefing in which the rebus stimulus was projected on the screen and subjects were asked to identify the pictured objects and subsequently to give five associations to the pictures of comb and raft, respectively. None of the subjects was able to identify the rebus level spontaneously. When explained, however, that these objects would represent a rebus and invited to solve it, all subjects were able to do so – but mostly only after being given numerous hints. After they had successfully solved the rebus, they were asked to give five associations to the rebus word (without a fight/struggle), as well.

## Data Analysis

In the following, the scoring of the free associations is described in more detail to illustrate the basic procedure. The same rules are applied to the scoring of dream reports and image descriptions.

As objective reference for the so-called *normative* scoring of the associations obtained after each awakening, we collected normative word association data to the rebus word (*kampflos*) and its components (*Kamm* and *Floß*) from a large group of people (reference group: *N* = 510). They were asked to give five associates to a list of pictures (including a picture of a comb and a raft) and words (including *Kamm*, *Floß*, and *kampflos*). By these means, we obtained extensive lists of associates for the key words, on the basis of which we could judge the appearance of stimulus-related words within the associates given by our subjects after each awakening. Associations were scored to belong to the secondary process *conceptual level* if they referred to the pictured objects of the comb and the raft; associations were judged to belong to the primary process *rebus level* in case they referred to the meaning “without a fight / struggle”^5^. Raters were blind to the respective condition (rebus or control) and sleep stage (REM or non-REM).

To depict the experimental effect of the rebus stimulus and to be able to compare the conceptual effect and the rebus effect with each other, difference scores were built by subtracting the scores of the control condition from those of the rebus condition. A positive score (and a higher mean rank) indicates a stronger experimental effect in the rebus than in the control condition, while a negative score indicates the reverse. To compare all three stages (wake, non-REM, REM) a Friedman two-way analysis of variance was performed on these difference scores and a Wilcoxon signed rank test for paired samples to depict differences between two stages each. Differences were considered significant at *p* < 0.05.

## Hypotheses

We hypothesize, that REM-sleep mentation follows the rules of the primary process, while non-REM-sleep and waking mentation are organized along more secondary process lines. Hence, we expect to find a stronger rebus effect as indicator for primary process thinking in associations (and dream reports and image descriptions) following REM-sleep awakenings; after non-REM-sleep awakenings and during wakefulness, however, more secondary process-like answers and associations on the conceptual level are expected.

## Results

### Sleep Quality

Since the experimental procedure involved six awakenings, it was very important that all participants had a normal sleep architecture and a healthy sleep. As could be seen in the baseline night, this was the case for all subjects. Average total sleep time was 7 h 27 min ± 58 min. All subjects fell asleep quite quickly (mean sleep onset after 9.9 ± 4.2 min) and displayed good sleep efficiency (90.9 ± 5.7%; a sleep efficiency above 85% describes a “healthy” sleep). Summarizing, and taking into account all additional parameters like periodic limb movements, apnea/hypopnea index and oxygen saturation, all subjects showed a healthy sleep architecture during the baseline night.

### Stimulus Detectability

As described above, a forced-choice detection experiment, based on Signal Detection Theory (Green and Swets, 1966) was run with each individual subject the morning after the last night in the sleep lab. The so-called criterion *c*, which reflects main response bias, was minimal (mean *c* = 0.02 ± 0.13). This indicates that subjects indeed distributed their “yes” and “no” responses evenly. Correct guesses (hit rates) ranged from 0.34 to 0.66 and incorrect guesses (false alarms) from 0.19 to 0.63. Mean *d*′, as measure of conscious perception (discrimination between stimulus and no-stimulus condition), was 0.07 ± 0.37 (-0.56 min, 0.80 max). The non-significant result of the one-sample *t*-test confirms our H0 hypothesis that *d*′ is at chance [*T*(19) = 0.847; *p* = 0.407]. This means, subjects could not detect a difference between stimulus and no-stimulus. Hence, we can indeed assume that stimuli were presented at the ODT for every single subject, and that there was no conscious awareness of the stimuli during the experiment.

### Free Associations

Our aim was to gain three non-REM-(stage 2) and three REM-sleep awakenings every night, which means 240 awakenings in total for the rebus and control night (120 non-REM and 120 REM awakenings). However, due to the unpredictable individual sleep behaviors, we obtained 226 awakenings in total – 115 non-REM-sleep awakenings (58 during the rebus night and 57 during the control night) and 111 awakenings from REM-sleep (57 during the rebus night and 54 during the control night). Therefore, we obtained 115 awakenings from the rebus night and 111 awakenings from the control night. All together, we obtained 1555 associations in the waking state, 3187 after awakenings from non-REM-sleep and 3195 after REM-sleep awakenings, which results in 7937 scorable associations in total. The average number of associations for wake, non-REM-, and REM-sleep for the rebus and the control night (corrected for the varying number of non-REM and REM awakenings) are displayed in **Table 1**. All associations were scored following the rules described above.

**Table 1:** Average number of wake, non-REM, and REM associations per subject in the rebus and control night (means ± standard deviation).

	Rebus night	Control night
Wake	41 ± 19	36 ± 24
non-REM	28 ± 18	27 ± 16
REM	30 ± 15	27 ± 16


*There were no statistically significant differences between nights or conditions.*

### Normative Scoring

For the *conceptual effect*, the difference between experimental and control night was significant (χ^2^ = 8.4; *p* = 0.015). The individual condition comparisons based on the Wilcoxon signed rank test for paired samples revealed a stronger conceptual effect in non-REM-sleep than in REM-sleep (*p* = 0.033). In the presleep waking stage, the effect was even stronger compared to REM-sleep (*p* = 0.002). The difference between wake and non-REM-sleep was not significant. With regard to the *rebus effect*, however, the differences between stages as revealed by the Friedman test were not significant. The results are summarized in **Table 2**.

**Table 2:** Normative and individual scoring for experimental conceptual and rebus effects in non-REM and REM free associations.

Effect type	Mean rank	Wake	Non-REM	REM
Conceptual	Normative	2.40^∗,†^	2.10^∗^	1.50
	Individual	2.26	1.97	1.76
Rebus	Normative	1.93	2.10	1.98
	Individual	1.95	1.76^∗^	2.29


*^∗^*p* < 0.05 significantly different from REM-condition; ^†^*p* < 0.005 significantly different from non-REM condition.*

### Individual Scoring

However, some important aspects need to be considered which presumably account for the fact that we did find the expected conceptual effect in non-REM-sleep, but no rebus effect in REM-sleep. One major reason might be the rebusword *kampflos* itself and its abstract, somehow paradoxical and potentially sensitive nature (see section “Discussion”). This is reflected by the fact that while all 510 people of the reference group gave only 47 different associates to *Kamm* and 74 associates to *Floß*, the amount of different *kampflos* associates is much larger (114). Furthermore, a closer look at the associates themselves reveals that people associate quite different words, feelings, and ideas to *kampflos* as opposed to *Kamm* and *Floß*. More specifically, while associates given to *Kamm* and *Floß* can be put in more or less one single category (e.g., “personal hygiene” for *Kamm* and “summery adventure” for *Floß*), this is not possible for the *kampflos* associates since they seem much more diverse [e.g., Friede (Engl.: peace), erschöpft (Engl.: exhausted), Hund (Engl.: dog), klug (Engl.: clever), Rom (Engl.: Rome)]. Apparently, there are no such prototypes of associations to *kampflos*, as there are to *Kamm* or *Floß*. As is illustrated in **Table 3**, the overlap between the associations given from the reference group and the associations obtained from one random subject in the debriefing, is much bigger within the comb and raft associations, as compared to the associations to the rebus word (without a struggle/ fight).

**Table 3:** Associations from the reference group vs. one subject (subject 11) for *Kamm*, *Floß*, and *kampflos.*

	Reference group	Subject 11
“Kamm” (Engl. comb)	Hair	*Hair*
	Brush	Head
	To comb	*Brush*
	Haircut	Man
	Hairdresser	*Haircut*

“Floß” (Engl. raft)	Water	*Water*
	Wood	*River*
	River	*Wood*
	Adventure	Huck Finn
	Ocean	*Adventure*

“kampflos” (Engl. without a struggle/ fight)	To give up	Abulic
	Peace	*Peace*
	Weak	Non-violent
	Coward	Verbal
	To lose	War


For this reason, it would be important to know the individual associations of each subject to the word *kampflos*, and score all associations of that single subject again, while taking the individual associates as reference. In fact, this was possible since we asked all subjects in the debriefing (see above) to give five associations to the *kampflos* stimulus. Therefore, we recounted the overlap between these debriefing associations on *kampflos* and the free assocations upon awakenings. This was done in the strictest way: though there was also thematic overlap (see further discussion), to avoid interpretation bias, only the literal words which were common to the associations upon awakening and to the debriefing were counted (objective count), and the count was divided by the total number of associations.

**FIGURE 2:**
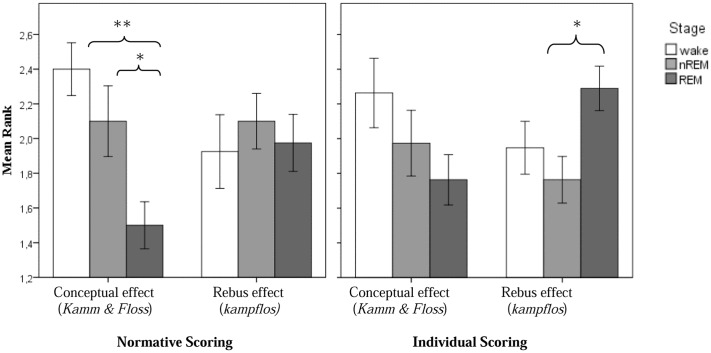
Experimental conceptual and rebus subliminal effect as a function of sleep stage (normative scoring, resp. individual scoring; ^∗^*p* < 0.05 and ^∗∗^*p* < 0.01).

The results are summarized in **Table 2**. Indeed, with regard to the *rebus effect*, the differences between stages as revealed by the Friedman test now become significant (χ^2^ = 8.4; *p* = 0.015) and individual group comparisons based on the Wilcoxon test for related samples revealed a significantly stronger *rebus effect* in REM-, as compared to non-REM-sleep (*p* = 0.013). The difference between REM-sleep and the presleep waking state and between non-REM-sleep and the waking state, respectively, were non-significant. However, the differences for the *conceptual effect*, though in the same direction as for the normative scoring, also became non-significant. In particular, there were no more differences between REM- and non-REM awakenings for the conceptual effect. **Figure 2** displays all experimental effects as function of stage.

### Dream Reports

After 162 of the total amount of 226 awakenings (115 non-REM- and 111 REM-sleep awakenings), we were able to obtain dream reports – 62 from non-REM-sleep stage 2 (54% of all non-REM awakenings), and 100 from REM-sleep (90% of all REM awakenings). This proportion of non-REM and REM dreams is typical, and reflects the numbers described in the literature (Nielsen, 1999). The length of the dream reports varied considerably between stages and subjects. Consistent with the literature, REM-sleep dream reports were much longer (94 ± 75 words in the rebus night and 89 ± 60 words in the control night) as compared to non-REM reports (24 ± 14 words in the rebus night and 52 ± 101 words in the control night; rebus condition: *p* = 0.002; control condition: *p* = 0.049). Following the scoring procedure described above yielded no significant differences. Neither the expected *conceptual effect* (predominance of *Kamm*- and *Floß*-related words in non-REM dream reports), nor the *rebus effect* (higher incidence of *kampflos* associations in REM dream reports) could be observed. Although the *conceptual effect* was slightly higher during non-REM-sleep, this difference remained non-significant (mean rank: 1.55 in non-REM versus 1.45 in REM). The same was true for the *rebus effect*: although we did find the expected tendency (stronger rebus effect in REM-sleep dream reports), these differences also remained non-significant (mean rank: 1.25 in non-REM versus 1.55 in REM).

### Image Descriptions

After 113 of the 226 awakenings (115 non-REM- and 111 REM-sleep awakenings), subjects were able to describe an image coming to mind immediately after the 4 min of free associations. 54 of these images were obtained after non-REM-sleep (47% of all non-REM awakenings) and 59 after awakenings from REM-sleep (53% of all REM awakenings). The scoring did not lead to any demonstrable conceptual or rebus effect. While the amount of *Kamm*- and *Floß*-related words in non-REM-sleep and REM-sleep image descriptions was identical (mean rank = 1.50), we found a slight increase in *kampflos*-related words within the REM-sleep image descriptions (mean ranks: 1.60 in REM versus 1.40 in non-REM) – however, without any significant effect.

## Discussion

The aim of this study was to test the hypothesis that REM- and non-REM-sleep and the waking state differ with respect to their prevailing way of mental organization and that these differences match the Freudian concept of primary and secondary process thinking. Therefore, we investigated the processing and solving of a subliminally presented rebus stimulus by analyzing the free associations, dream reports and image descriptions given by the subjects in the waking state and after REM- and non-REM-sleep awakenings during the following night.

### Primary and Secondary Process Effects

Using the normative scores, we found a stronger conceptual effect in free associations after awakenings from non-REM-sleep as compared to after awakening from REM-sleep, but no rebus effect. Using the individual debriefing socres, there was no difference in conceptual effect between both stages but we found a stronger rebus effect within free associations after REM-awakenings as opposed to after non-REM-awakenings. A tendency for a stronger rebus effect after REM-sleep awakenings and a stronger conceptual effect after non-REM-sleep was also found for dream reports, but the effects remained statistically non-significant. Finally, no rebus or conceptual effect was found within image descriptions. In summary, we had no main result in which we were able to replicate at the same time a stronger conceptual effect after non-REM awakenings and a stronger rebus effect after REM-awakenings, as was found in the original study by Shevrin and Fisher (1967). When we used the same scoring methodology as theirs, a robust conceptual effect was replicated but the rebus effect remained non-significant; however, a rebus effect emerged by using each subject’s individual associations.

In that light, it is interesting to explore how the “penny”-rebus differed from the rebus of the present study. First, it is clear that for “penny” both the composing words (“pen” and “knee”) and the final rebus word (“penny”) are common everyday concrete objects. This is also true for *Kamm* and *Floß* – although a raft is already less common than a pen, a knee or a comb. But, although we took great effort in finding a suitable rebus words of which both composing elements were easily illustratable and inconclusively identifiable^6^, the resulting rebus word *kampflos* is an abstract and furthermore paradoxal quality, namely: to overwin without a fight or a struggle. Thereby, it strongly depends on whether one focuses on the Kampf-aspect and thus associates rather negative-toned words like “war,” “blood,” or “enemy” – or whether one concentrates more on the positive part (*without* a fight) and thus gives associates like “peace,” “harmony,” or “friendship.” Moreover, the word *kampflos*, containing also the word *Kampf*, might have a more than average emotional connotation in a German population and might have induced subjective conflict, which explains why it might more easily trigger personal associations than neutral common words^7^. The present results based on the individual associations, therefore, agrees with the idea that the nature and the strength of the subliminal effect depends on the subject’s personal history and individual representations and experiences, as already observed by the authors of the first dream-rebus report (Fisher, 1960; Shevrin et al., 1996). However, it might be that, in general, a non-conflictual word is an unrealistic idea, as e.g., the word ‘penny,’ referring to the psychodynamic important and conflictual theme of ‘money’ (as well as, phonologically, hinting to another psychodynamic important and conflictual theme, namely through the consonance with the neighboring word ‘penis’) is also not straightforwardly neutral and non-conflictual. However, at difference with the penny-rebus, the conflicts induced by the *kampflos*-rebus, are not referring to universal, human condition nodal points, but rather, to contingent and personal history elements^8^.

Secondly, the robust conceptual effect obtained with the normative scorings diminished with the individual scorings. However, the present results also do not plead for a reversal of the effect, but simply that when using the more idiosyncratic material, more associations can be picked up, and thereby the difference subsides and is no longer significant. This, then, might be a suggestion for the fact that both conceptual and rebus REM-associations are more idiosyncratic than non-REM-associations, which agrees with other observations (see section “Introduction”). In other words, the clarity of the results suffers from the fact that idiosyncracy is thought to play an important role with the present rebus: therefore, the rebus results can only be revealed with the idiosyncratic associations. However, idiosyncrasy as such is a primary process effect (Gabbard et al., 2012, p. 473), for the more the associations are personal, the less they are rational. In the same logic, idiosyncracy at the level of the conceptual effects, possibly contaminates a supposedly secondary process measure with primary process elements, and thereby leads to revealing more conceptual effects after REM-sleep too. The rebus effects, however, are always implying primary process mentation as any corresponding associations on *kampflos* must obligatorily have implied a rebus condensation first, since the word *kampflos* was not presented as such. Taking all this together, these results by and large support the proposed double dissociation with more secondary processing during wake and after non-REM state versus more primary processing after REM-state, though, admittedly, our results are less straightforward and more complex than the results of the first study.

It has been reported frequently before that dreaming and associative creativity are correlated (e.g., Stickgold et al., 1999; Mazzarello, 2000), but the underlying mechanisms, such as sleep stages have less frequently been explored. However, when they were, the results are coherent with the present ones. For example, Walker et al. (2002) compared the performance of 16 subjects on a test of cognitive flexibility using anagram word puzzles (e.g., ‘OSEOG’ = ‘GOOSE’) following awakenings across the night. It was found that REM awakenings provided a significant 32% advantage in the number of anagrams solved compared with non-REM awakenings. Likewise, Cai et al. (2009), using a Remote Associates Test, requiring the subject to relate three words drawn from mutually remote associative clusers (e.g., COOKIES, SIXTEEN, HEART) in order to find a fourth word that could serve as an associative link between these three words (e.g., SWEET), found that compared with quiet rest and non-REM-sleep, REM-sleep enhanced the formation of associative networks and the integration of unassociated information. We propose that these results, although not interpreted in a psychoanalytic framework, can easily fit in the proposal of REM-sleep favorising primary process mentation.

### The Experimental Protocol

One might question why the stimulus needs to be presented subliminally for the expected effects to occur or whether we would have found the same effects if the rebus stimulus had been presented supraliminally. In fact, Fisher (1960) was able to demonstrate that supraliminally presented stimuli are incorporated into subsequent dreams, as well. However, they reappeared in a conceptual, secondary process way, whereas primary process transformations were only observable after subliminal exposure. Similarly, Shevrin and Luborsky (1958, 1961) and Shevrin and Fritzler (1968) found that primary process effects within free associations could be obtained after the subliminal presentation of the penny rebus, but not after supraliminal exposure. Apparently, an unconscious re-working of the respective stimulus is necessary to result in primary process transformations, and when processed supraliminally, stimuli are structurally submitted to an inhibitory defense process, which refrains, primary process treatment (Freud, 1915/1959; see also Bazan, 2007a,b, 2017). The present findings are remarkable since main effects at the ODT are very rarely found (Snodgrass et al., 1993; Klein Villa et al., 2006).

Another question is why the subliminal rebus stimulus of all possible stimuli which the subject registers during the day, should enter the mental processing during the night. The results might mistakenly induce the idea that the subliminal rebus induced dream material which stands out compared to other potential dream themes – such as those referring more clasically to wish-fulfilments and day residuals. However, it is important to remind the reader that we only could reveal the reference of the dream materials to the rebus stimulus by comparing the participant’s associations with association norms *to the rebus* in experimental and control conditions. Nonetheless, we think that the reappearance of the stimulus material in the dreams might have been helped with the particular transference conditions of the experiment. Introducing the psychoanalytic concept of transference into the experimental situation and focusing on the relationship between the subject and the experimenter, Fisher (1954) assumed that subjects who are instructed to record their dreams after the presentation of some stimulus might interpret this instruction as an indirect command to dream about the presented stimulus. This claim is based on the psychoanalytic view of the experimental situation “as one in which the relationship between the experimenter and the subjects leads the latter to have a dream structured around several unconscious wishes activated by the total experimental situation” (Dixon, 1971). Hence, dreams obtained within subliminal experiments might be conceived as transference dreams. Indeed, Fisher was able to demonstrate in his experiments that subjects who had a good positive transference to the experimenter showed greater subliminal effects (Fisher, 1960). For all these reasons, it could be assumed that it is the transference to the experimenter which might have facilitated the finding of the results. In this respect, it is interesting to note that the study reported here required a lot from the subjects. They were willing to spend three nights in the sleep lab on three successive weekends and agreed to be woken up six times per night. Despite these demanding requirements, none of the subjects quitted the study, which could plausibly be explained by a good transference to the experimenter. Furthermore, the nature of the study caused quite intimate situations. Subjects were sitting in their pajamas for about 1 h while the cables were attached. They were observed in a very private and unprotected state: during sleep. They were asked to give insight into very personal details: their dreams. Hence, the subjects allowed the experimenter to enter a very private sphere. Because they did so, we could assume that they fully trusted the experimenter – which again can be explained with a positive transference.

The expected effect was mainly carried by the free associations, confirming its retrieval capacity of unconscious processes and subliminal effects. In contrast to the associations, dream reports failed to show significant effects, although the expected tendency could be observed. By using the method of free associations, and assuming a certain carry-over effect from the preceding sleep stage on wakefulness (Nielsen, 2000), however, we assume we were able to get closer to the process of dreaming rather than to its end product. This is, in fact, not very different from Freud’s findings in *The interpretation of dreams* (Freud, 1900/1953). In agreement with the present findings, the dream reports in Freud’s text are themselves often short, factual and to some degree perplexifying. All the links of interest to the themes which presumably preoccupied the dreamer, come from the free associations on the basis of the manifest dream, and it was in this mycelium^9^ of free associations that Freud discerned primary process manifestations, such as displacements and condensations. We therefore think that it is logical that our findings are first and foremost in the free associations, as, even if these are ‘free,’ they are in time immediately next to the dream reports and therefore can be easily understood as influenced by the dream material.

We also failed to demonstrate the expected subliminal conceptual and rebus effect within image descriptions. This could be due to the fact that images are primarily visual in nature, whereas the measured effects of this experiment are largely verbal. Hence, the verbal influences investigated in this study might not be detectable in the images as a primarily visual experience. This was also the case in the original study of Shevrin and Fisher (1967). Therefore, it simply may be that the static image material is not enough of a dynamical support for signification effects (either primary or secondary) to show up. All in all, these findings match the results of the study of Shevrin and Fisher (1967). Indeed, these authors also found a stronger rebus effect in associations obtained after REM-sleep awakenings and a stronger conceptual effect within non-REM-sleep associations, but no effects whatsoever in dream reports or image descriptions.

## Limitations

As discussed, one major impediment was the special nature of the *kampflos*-rebus stimulus, which implied some problematic aspects, such as the inducing of a high variety of personal associations, as discussed. Another limitation concerns the sample size: although we studied twice as many subjects as in the original study, a sample size of *N* = 20 still limits the degree to which the results can be generalized. Furthermore, the proportion of male to female subjects (5:15) might induce gender biases in the results.

Finally, with the rebus method only a very specific aspect of primary process thinking was probed. For example, Chabert (2012, p. 243) uses a very wide range of different elements to evaluate primary processes in Rorschach protocols, namely: “Prolixity, the multiplication of responses, abundant, full, confused verbalization then give rise to oddities and discrepancies. The production can then be very important, poorly ordered, provided in a hurry, the speech is fuzzy, vague, indeterminate.” She also proposes to highlight the presence of responses the location of which is arbitrary or ill-defined, inconsistent, as well as the presence of responses reflecting a poor quality of formal control and perception skids (Chabert, 2012, p. 243; see also Debroux et al., 2009). Obviously, in dream material we also could have scored deviations from reality and delusional elements, unreal characters, characters with a major persecuting connotation, as well as archaic personas, dominated by omnipotence, dangerousness, or destructiveness. All these elements are also valuable parameters and which could be used in much less complicated protocols, simply measuring these elements in standardized ways and without prior (subliminal or supraliminal) stimulation upon REM- and non-REM awakenings. However, we might add that (1) this type of research is then very close to studies probing for bizarreness and indeed finding higher bizarreness scores in REM- as compared to non-REM-dreams (for references, see higher); (2) the rebus method is much closer to Freud’s original counter-intuitive proposition in *The Interpretation of dreams* (Freud, 1900/1953), where he explains that dreams are characterized by rebus word puzzles and condensations, which for their interpretation – i.e., for the recovery of the latent dream material – should be read on that literal level, as opposed to a metaphorical reading. Finally, by using the rebus method and association norms for scoring, we avoid the need for any kind of interpretation of the dream materials (which would be required by the above-mentioned form- and content parameters), thereby circumventing Grunbaum’s (1984) critique of circularity in psychoanalytic research. As mentioned above, only the literal words, which were common both to the associations upon awakening and to the debriefing associations, were counted, disregarding any element of thematic overlap, such as to avoid any interpretation bias in the strictest way.

## Conclusion

The present subliminal priming results are indicative of the fact that the subliminally presented stimulus was processed during sleep and that, furthermore, its rebus level could be read unconsciously and appeared in the free associations obtained after REM-sleep awakenings. This latter result supports the relevance of the psychoanalytic method of free associations to trace back unconscious material.

To summarize, the results show a rebus effect, as an index for primary process thinking, within the free associations following REM-sleep awakenings when scored with the individual associations. They also show a strong conceptual effect, as an index for secondary process thinking, within the free association obtained after non-REM-sleep awakenings when scored with the normative associations. We did not obtain this double dissociation (more rebus after REM versus more conceptual after non-REM) within one scoring system (individual or normative), which is a weakness of the present results. However, the general tendency remains when using the individual norms, which are the most suitable in the light of the particularity of the *kampflos* rebus. By scoring the conceptual effects with the individual (instead of the normative) associations on *Kamm* and *Floß*, we included both secondary process conceptual (meaning) and idiosyncratic primary process associative hits into the total count for “conceptual effect,” thereby unduly increasing the number of “conceptual hits” after REM awakenings and diminishing the difference in conceptual effect between REM and non-REM. For this reason, our results by and large replicated the original Shevrin and Fisher (1967) results and our findings support the idea that REM- and non-REM-sleep mentation differ with regard to their prevailing mental organization: while REM-sleep is characterized by primary process thinking, non-REM-sleep mentation follows the rules of the secondary process.

## Ethics Statement

The protocol was approved by the ethics committee of the University of Bremen. All subjects gave written informed consent in accordance with the Declaration of Helsinki.

## Author Contributions

JS planned and conducted the research, data-analysis, interpretation and write up. AB helped at all stages of the research from funding application, to design, over data-analysis to write up. She was not involved in data gathering as such. SH oversaw the sleep-experiments and added sleep expertise. SA assisted with the research, especially data-analysis and helped with write up. HS oversaw the research, from design to data-analysis and interpretation.

## Conflict of Interest Statement

SH received financial support from UCB Pharma, Mundipharma, Bayer HealthCare, Merz, Allergan, Kappernagel & Menßen, Boehringer Ingelheim, Bristol-Myers Squibb/Pfizer, Abbvie, Bial – all not related to the submission of this manuscript. The other authors declare that the research was conducted in the absence of any commercial or financial relationships that could be construed as a potential conflict of interest.

## Footnotes

1 Polysemy is the coexistence of many possible meanings for a word or phrase.

2 As Freud stated in the *Interpretation of Dreams*: “The dream-content is, as it were, presented in hieroglyphics, whose symbols must be translated, one by one, into the language of dream-thoughts. It would of course, be incorrect to attempt to read these symbols in accordance with their values as pictures, instead of in accordance with their meaning as symbols. For instance, I have before me a picture-puzzle (rebus) – a house, upon whose roof there is a boat; then a single letter; then a running figure whose head has been omitted, and so on. As a critic, I might be tempted to judge this composition and its elements to be non-sensical. A boat is out of place on the roof of a house, and a headless man cannot run; the man, too, is larger than the house, and if the whole thing is meant to represent a landscape the single letters have no right in it, since they do not occur in nature. A correct judgment of the picture-puzzle is possible only if I make no such objections to the whole and its parts, and if, on the contrary, I take the trouble to replace each image by a syllable or a word which it may represent by virtue of some allusion or relation. The words thus put together are no longer meaningless, but might constitute the most beautiful and pregnant aphorism. Now a dream is such a picture-puzzle, and our predecessors in the art of dream-interpretation have made the mistake of judging the rebus as an artistic composition. As such, of course, it appears non-sensical and worthless” (Freud, 1900/1953, p. 169–170).

3 According to Freud (1895/1950, 1900/1953, 1915/1959), the dynamic unconscious is part of the unconscious and the result of repression. Repression implies that the representational or idea part of an experience is separated from its affective charge – the representation, devoid of its investment, is thereby rendered dynamically unconscious.

4 In other words, the dream does not use style figures to picture prozaic matters, but, the other way around, uses prozaic presentations to refer to abstract expressions. In this, dreams are the reverse of novels. One striking example of this reversal is that in novels real-life characters are introduced as themselves but with different names and different identity attributes, while in a dream real-life characters are introduced as totally different persons, bearing however, the same names and/or identity attributes. In short, dreams do not deal into poetry, but are to be read as the most prozaic descriptions possible of metaphorical, abstract, style and grammar figures. Dreamers usually engage in the interpretations of their dreams by taking them metaphorically: in this, they omit to engage into literal associations upon the dream materials, and are thereby in solidarity with their defenses.

5 A scoring manual is available on request from Steinig (2011, pp. 54–57 and Annex F) in which the rules for using these measures are fully described.

6 It was definitely a problem to find a suitable German rebus (see Steinig, 2011, p. 64). Involved in the search for a rebus were several linguistic institutes of German universities, who were asked to pass on this request to their students. Additionally, a broad public was addressed by an online competition in which a prize was promised for the best rebus. The *kampflos*-rebus was the only one which fulfilled all requirements, even if still being far from the perfect rebus.

7 To illustrate this with one example: Subject 19, when awakened from the REM-stages of the experimental night gives the following associations: “Angsteinflößend [frightening] – Pulsierend [pulsating] – Klopfen [beat] (…) Angst [anxiety] (….) Blut [blood] – Unruhe [restlessness] (…) Fertig [done] – (…) Memory [memory] – Rot [red]” while nothing of the kind followed awakenings from non-REM (e.g., “Müde [tired] – Dösig [drowsy] – Kein Anhaltspunkt [no clue] – Schwer [heavy] – Augen [eyes] – Gedanken [thoughts] – Kreisen [revolve]) or from REM on control nights (e.g., Leicht [light] – Pochen [throb] – Außen [outside] – Innen [inside] – Tiefe [Depth] – Benommen [dazed] – Wanken [waver]”).

8 As the debriefing associates were given in the same study which is to measure a possible experimental association effect, we have to exclude the possibility that our results were due to an inverse direction of the association: namely, the possibility that the associations at nightly awakenings contaminated the debriefing associations upon the morning of the last night. Let us first repeat how and at what stage of the protocol, these associations were obtained: all participants slept three nights in the sleep lab, a baseline night (which was always the first), an experimental night (preceded by the rebus priming) and a control night (preceded by the blank slide). The order of the experimental and the control night was alternated over the participants. As the debriefing with the personal associations always came in the morning of the last night, for half of the participants this was after the experimental night and for the other half this was after the control night. In other words, even if it is reasonable to think that the free associations given during the night influenced the free associations during the debriefing the following morning, thereby inducing artificial ‘hits,’ this potential effect will be there for both the experimental and the control condition. As the results are calculated by substracting control from experimental, any spurious effect of the sort is thereby neutralized. Moreover, upon verification, there were not more ‘hits’ after the last night, independent of condition, than after the second night.

9 Freud states that “the dream-wish arises like a mushroom out of its mycelium” (Freud, 1900/1953, p. 525), thereby introducing the metaphor of the ‘mycelium’ to describe the dense knot of associations in a dream connecting the elements of the dream, in the same way the mycelium is the network of subterranean fibers that connect a group of mushrooms. Above ground we see separate elements, but below ground we see evidence of connected themes.
